# Sulforhodamine 101, a widely used astrocyte marker, can induce cortical seizure-like activity at concentrations commonly used

**DOI:** 10.1038/srep30433

**Published:** 2016-07-26

**Authors:** Rune Rasmussen, Maiken Nedergaard, Nicolas Caesar Petersen

**Affiliations:** 1 Center for Translational Neuromedicine, University of Rochester Medical Center, Rochester, New York 14642, USA; 2 Center for Basic and Translational Neuroscience, University of Copenhagen Faculty of Medicine, 2200 Copenhagen N, Denmark; 3 Department of Nutrition, Exercise and Sports, University of Copenhagen, 2200 Copenhagen N, Denmark; 4 Department of Neuroscience and Pharmacology, University of Copenhagen, 2200 Copenhagen N, Denmark

## Abstract

Sulforhodamine 101 (SR101) is a preferential astrocyte marker widely used in 2-photon microscopy experiments. Here we show, that topical loading of two commonly used SR101 concentrations, 100 μM and 250 μM when incubated for 10 min, can induce seizure-like local field potential (LFP) activity in both anaesthetized and awake mouse sensori-motor cortex. This cortical seizure-like activity develops in less than ten minutes following topical loading, and when applied longer, these neuronal discharges reliably evoke contra-lateral hindlimb muscle contractions. Short duration (<1 min) incubation of 100 μM and 250 μM SR101 or application of lower concentrations 25 μM and 50 μM of SR101, incubated for 30 and 20 min, respectively, did not induce abnormal LFP activity in sensori-motor cortex, but did label astrocytes, and may thus be considered more appropriate concentrations for *in vivo* astrocyte labeling. In addition to label astrocytes SR101 may, at 100 μM and 250 μM, induce abnormal neuronal activity and interfere with cortical circuit activity. SR101 concentration of 50 μM or lower did not induce abnormal neuronal activity. We advocate that, to label astrocytes with SR101, concentrations no higher than 50 μM should be used for *in vivo* experiments.

The fluorescent indicator dye sulforhodamine 101 (SR101) is a water-soluble compound, widely used for labelling of astrocytes for *in vitro* and *in vivo* 2-photon microscopy experiments^1^,^2^,^3^,^4^,^5^ (see Supplementary Table S1) and recently of oligodendrocytes^6^.

When used for *in vivo* microscopy, SR101 is routinely applied topically to the exposed cortex, from where it is taken up by astrocytes^7^ and diffuses through the astrocyte syncytium via gap junctions, brightly labelling astrocyte somata, processes and perivascular end-feet^8^. SR101 has in particular been used in combination with loading of chemical Ca^2+^ indicators such as Oregon Green 488 BAPTA-1 AM to unambiguously distinguishing between neuronal and astrocytic Ca^2+^ signals^1^,^3^.

We and others have previously described bioactive effects of SR101 on excitatory neuronal activity^9^,^10^. In hippocampal slices, pyramidal CA1 neurones showed long-term potentiation of intrinsic excitability and enhanced synaptic efficacy following a short period of SR101 application^9^. Intra-neuronal recordings indicated that the increased excitability was a consequence of lowering the threshold for evoking action potentials and was observed following application of just 1 μM SR101 for ten minutes.

The original publication by Nimmerjahn and colleagues reported that SR101 does not induce phototoxicity over hours, days and weeks^8^. Here we have combined SR101 loading with recordings of the local field potential (LFP) during the labeling period to interrogate if SR101 causes changes in cortical neuronal activity *in vivo*.

We explored the effects of topical SR101 application on neuronal activity in layer II of primary sensori-motor cortical areas (S1-M1) of adult anaesthetized or awake mice. We show that spontaneous seizure-like LFP activity develops within less than ten minutes following cortical exposure to 100 μM SR101 for ten minutes, a commonly used dosage for astrocyte labelling (see Supplementary Table S1). This S1-M1 seizure-like neuronal activity was accompanied with a reduction in low-frequency power, and simultaneous increase in higher-frequency power in the LFP. When exposing the cortex to 250 μM SR101 for ten minutes the seizure-like activity developed within five minutes and ceased after about 45 minutes. In addition, we show that SR101-induced seizure-like activity in S1-M1 is sufficiently potent to drive a motor output, recorded as compound action potentials in the sciatic nerve of the contra-lateral hindlimb or by observation of movement. Finally, we show that 25 μM and 50 μM SR101 application, incubated for 30 or 20 minutes respectively, or 100 μM and 250 μM incubated for one minute, does not induce abnormal cortical LFP activity, and may thus be considered as safe dosages.

## Results

### SR101 can induce seizure-like LFP activity in anaesthetised mice

**Figure 1:**
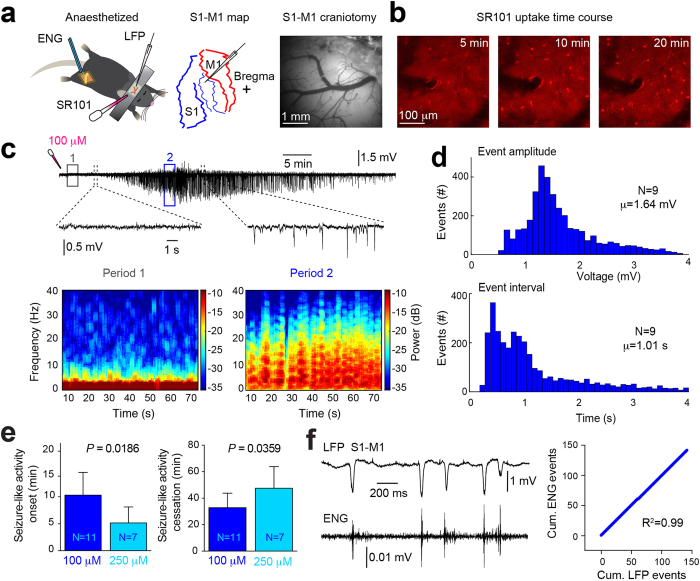
SR101 can induce seizure-like activity in anaesthetized mice. (**a**) Experimental setup and craniotomy above primary sensori-motor cortex (S1-M1). (**b**) Astrocytic SR101 uptake time course. (**c**) Upper, LFP during development of seizure-like activity in S1-M1 following 100 μM SR101 loading (10 min incubation). Lower, frequency power spectrograms from period 1 and 2 from LFP above. (**d**) Histograms showing individual seizure-like event amplitudes and intervals. (**e**) Graph showing dose-dependency of seizure-like activity onset and cessation. Statistical difference was assessed using an unpaired Welch’s *t*-test. (**f**) Left, simultaneous S1-M1 LFP and ENG following 250 μM SR101 application (10 min incubation). Right, graph showing cumulative number of ENG events as a function of seizure-like LFP events. Correlation was assessed using a Pearson’s correlations test.

We first recorded LFPs in S1-M1 of ketamine (120 mg kg–1) and xylazine (10 mg kg–1) (KX) anesthetized mice while topically applying 100 μM or 250 μM SR101 for 10 minutes (Fig. 1a). In a subset of experiments, we included recordings of the electroneurogram (ENG) from the contra-lateral sciatic nerve, to provide a measure of cortical-evoked motor output (Fig. 1a), in addition video recordings were obtained to document the motor activity (see Supplementary Videos S1–S3). Following topical application of SR101, astrocytes exhibited a time-dependent increase in SR101 fluorescent intensity, with a marked increase occurring ~10–20 minutes following application (Fig. 1b). Simultaneous LFP recordings in S1-M1 layer II revealed that large synchronized potentials, mimicking epileptic seizure activity in terms of individual event amplitude^11^,^12^,^13^ and event interval^14^,^15^,^16^, appeared within 5–10 minutes following topical 100 μM SR101 application (Fig. 1c).

The seizure-like activity was correlated with a reduction in low-frequency power (1–4 Hz) and a slight increase in higher-frequency power (12–30 Hz) (Fig. 1c and Supplementary Fig. S1). Such increases in higher-frequency power bands have previously been showed in animal models using kainate or optogenetics to evoke epileptic acivity^13^,^17^.

Similar changes in cortical LFP activity were not recorded under control conditions (see Supplementary Fig. S2). An example of a non-filtered recording displaying the artefacts evoked by application and wash out of SR101 10 min later is provided in Supplementary Fig. S3. The seizure-like activity displayed a gradual build-up in amplitude, similar to prevously shown during 4-AP-induced epilepsy^15^, with the individual seizure-like events exhibiting an average amplitude of 1.64 ± 0.67 mV and average event interval of 1.01 ± 0.88 s (Fig. 1d), similar to previous reports using caged 4-AP, kainate or optogenetics to evoke seizures^11^,^12^,^13^. The term “seizure-like” was chosen based on the LFP’s resembles to previously published LFP activity during epileptiform activity (*i.e.* event amplitude, event frequency and 12–30 Hz LFP power band increase), induced by commonly used epilepsy models such as kainate, 4-AP or kindling^12^,^15^,^18^. However, due to the lack of mechanistic insight to what is triggering the abnormal neuronal discharges following SR101 application, we chose “seizure-like”, as opposed to simply “epileptiform” or “seizure”.

In many publications, SR101 is applied topically at concentrations ranging from 1 μM to 1 mM (see Supplementary Table S1), most commonly between 100–300 μM^4^,^15^,^19^,^20^, and incubated for 1–60 minutes (see Supplementary Table S1), often for 10–30 minutes^15^,^20^,^21^. SR101 induced a dose-dependent onset and cessation of the cortical seizure-like LFP activity (Fig. 1e); at a loading concentration of 250 μM (incubated for 10 min), seizure-like activity appeared at 5.16 ± 3.41 min, but first 10.31 ± 5.1 min after application of 100 μM (incubated for 10 min) SR101 (Unpaired Welch’s *t*-test, t = 2.819 df = 9.771, *P* = 0.0186). The seizure-like LFP activity disappeared after 47.50 ± 19.41 min and 32.83 ± 12.38 min following SR101 application, for 250 μM and 100 μM SR101, respectively (Unpaired Welch’s *t*-test, t = 2.292 df = 15.91, *P* = 0.0359). Muscle twitches occurring in the contra-lateral hindlimb was observed ~20 minutes following topical SR101 application (see Supplementary Videos S1–S3). To assess whether the movement of the contra-lateral hindlimb originated from S1-M1, we obtained simultaneous LFP recordings in S1-M1 and ENG from the contra-lateral sciatic nerve (Fig. 1a). These recordings showed that S1-M1 SR101 application (250 μM, ten minute incubation) correlated with arrival of compound action potentials in the sciatic nerve, and that individual seizure-like LFP events and ENG compound action potential events were almost perfectly matched in a 1:1 ratio (Fig. 1f) (Pearsons’s correlation test, R^2^ = 0.99). An example of a longer recording is provided in Supplementary Fig. S4. A compound structurally similar to SR101, but with a SO_3_H group replaced by SO_2_NHNH_2_^22^, is Texas Red, also used to label astrocytes^8^,^23^. Opposite to SR101 did topical Texas Red application (100 μM and 500 μM, 15 min and 10 min incubation time, respectively) not evoke any seizure-like LFP activity (see Supplementary Fig. S6). Taken together, these experiments show that topical SR101 application can have pronounced effects on cortical neuronal activity when applied at commonly used loading concentrations, *i.e.* 100 μM and 250 μM, in anaesthetized mice.

### SR101 can induce seizure-like LFP activity in awake mice

Anesthesia is known to have anti-epileptic effects^24^ and anesthesia markedly changes the membrane potential dynamics of neurones^25^. Moreover, *in vivo* 2-photon microscopy experiments are increasingly based on analysis of awake animals^4^.

**Figure 2:**
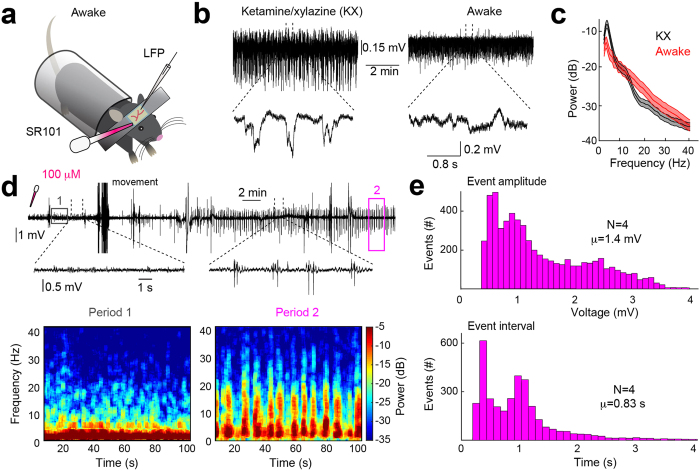
SR101 can induce seizure-like activity in awake mice. (**a**) Experimental setup. (**b**) LFP recordings from the same mouse during ketamine/xylazine (KX) anaesthesia and wakefulness. (**c**) Frequency-domain spectra showing change in power distribution between KX anaesthesia and awake LFP from (**b**). (**d**) Upper, LFP recording showing development of seizure-like activity in primary sensori-motor cortex following 100 μM SR101 loading (10 min incubation). Note that movement artefacts have not been removed from the LFP. Lower, frequency power spectrograms from period 1 and 2 from LFP above. (**e**) Histograms showing individual seizure-like activity event amplitudes and intervals.

We therefore analyzed the effects of SR101 application in awake non-anaesthetized mice (Fig. 2a). Following preparation of the cranial window the mice were allowed to wake up from the anesthesia for 1 hour and wakefulness was then confirmed by decreased 1–4 Hz power and increased 12–30 Hz power in the LFP^26^ as well as by visual confirmation (Fig. 2b,c and Supplementary Fig. 7a–c). When we recorded LFPs in S1-M1 of awake mice we again observed large synchronised seizure-like potentials occuring following 100 μM (10 min incubation) SR101 application (Fig. 2d). Note that movement artefacts were not removed from the LFP trace shown in Fig. 2d. This seizure-like activity was correlated with a drop in low-frequency power (1–4 Hz) and an increase in higher-frequency power (12–30 Hz) (Fig. 2d and Supplementary Fig. S1) similar to seen in Fig. 1b and previous epilepsy publications^13^,^17^. The individual seizure-like events in awake mice exhibited an average amplitude of 1.4 ± 0.82 mV and average event intervals of 0.83 ± 0.75 s (Fig. 2e). Thus, the average seizure-like event amplitude was 0.24 mV smaller (Unpaired Welch’s t-test, t = 1.632 df = 8.605, *P* = 0.1387) and the event interval 0.16 s shorter (Unpaired Welch’s t-test, t = 2.181 df = 9.934, *P* = 0.0543) than in anaesthetized mice. Overall, this experiment expands the finding that SR101 evoke seizure-like cortical activity to awake animals, potentially making it useful as an epileptogenic agent for future *in vivo* experiments investigating cortical epilepsy, conditioned that SR101’s mechanism of action will be revealed in future experiments.

### Lower concentrations or shorter incubation time does not evoke abnormal cortical LFP activity

**Figure 3:**
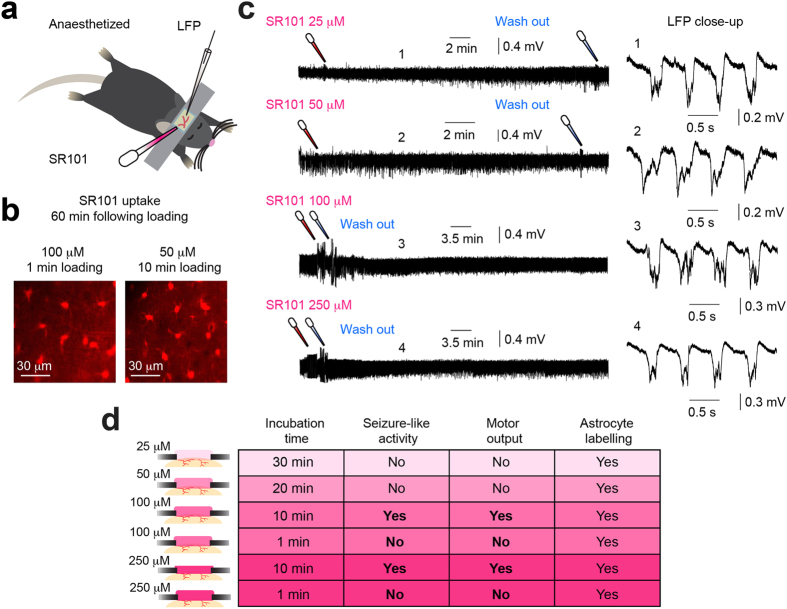
Lower SR101 concentrations or shorter incubation time does not evoke abnormal cortical LFP activity. (**a**) Experimental setup. (**b**) Astrocytic SR101 uptake following cortical 100 μM (1 min incubation) or 50 μM (10 min incubation) SR101 application (images acquired 60 min following loading). (**c**) *Left*, LFP recordings following 25 μM (30 min incubation), 50 μM (20 min incubation), 100 μM (1 min incubation) or 250 μM (1 min incubation) SR101 application. *Right*, close-up of LFPs shown on the left. (**d**) Summary table showing main findings.

The original publication by Nimmerjahn and colleagues, showing the ability of SR101 to label cortical astrocytes *in vivo*, recommended using 25–100 μM SR101 and incubation for one to five minutes^8^. However, from Supplementary Table S1 provided in the supplementary material, it appears that a large fraction of publications use concentrations higher than this (see Supplementary Table S1). In Figs 1 and 2 we show that topical loading of 100 μM and 250 μM SR101 (10 min incubation) may induce abnormal cortical neuronal activity, an effect that ideally is undesireable. Thus, in the final experiment we tested the effect of lower concentrations, *i.e.* 25 μM and 50 μM, of SR101 and concentrations of 100 μM and 250 μM incubated for one minute on cortical neuronal activity. We observed bright labelling of cortical astrocytes using either 100 μM (1 min incubation) or 50 μM (10 min incubation) SR101 (Fig. 3b,d). Contrary to the previous experiments, we did not observe any abnormal LFP activity in the anaesthetised cortex following loading, despite leaving the SR101 on for 30 min and 20 min for 25 μM and 50 μM, respectively (Fig. 3c–d and Supplementary S5). Non-filtered recordings displaying the application and wash out induced artefacts, together with power spectrograms, are provided in Supplementary Fig. S5. Thus, from these experiments it appears that concentrations of 50 μM, or less, SR101 does not induce abnormal cortical neuronal activity, but still sufficiently labels astrocytes (summarised in Fig. 3d).

## Discussion

Here we report that SR101, a widely used fluorescent dye for specific labelling of astrocytes, possess potent bioactive effects able to change cortical neuronal activity *in vivo* following topical loading.

Currently the mechanism for how SR101 triggers the observed change in cortical neuronal activity is unknown. The two most likely hypotheses may be that: (1) SR101 has a direct effect on cortical neurones, or (2) SR101 has an indirect effect on neuronal activity via an astrocyte-mediated mechanism. We have previously shown, that in hippocampal slices SR101 induces a direct effect on pyramidal neurone membrane structures, leading to a reduction in action potential firing threshold, and a long-term increase in neuronal excitability and synaptic efficacy^9^. This finding is in agreement with a similar experiment performed in hippocampal slices^10^. Here the authors showed that SR101 markedly increased the excitability of pyramidal neurones as well as induced monosynaptic long-term potentiation of synaptic efficacy in hippocampal area CA1. These effects developed already after five minutes following 100 μM or 25 μM SR101 bath application^10^. This time course agrees with our finding that SR101-induced seizure-like LFP activity develops within five to ten minutes following topical loading, depending on the applied concentration (Fig. 1). This rather rapid time course favours a direct SR101-mediated effect on neurones. We observed that cortical astrocytes starts to appear bright under the 2-photon microscope within ten to twenty minutes following 100 μM SR101 application (Fig. 1), similar to what Nimmerjahn and colleagues reported in the original publication^8^. If the SR101-triggered effect on neuronal LFP activity was via an astrocyte-dependent intra-cellular mechanism, one might expect that SR101 uptake into cortical astrocytes preceded that of LFP changes; this did not seem to be the case. Thus, it appears more likely that SR101 has a direct effect on neurones while the concentration is still relatively high in the extracellular environment. This may also be supported by the striking similarity between the seizure-like LFP activity presented here (Fig. 1), and previously published epileptiform activity induced *in vivo* by application of the compound 4-AP, targeting neuronal potassium channels^15^. However, recent work have suggested astrocyte involvement in the development of epileptic activity^15^,^27^,^28^, and it is conceivable that SR101-induced astrocyte signalling occurs following uptake and contributes to the observed seizure-like LFP activity. Similar, a possible oligodendrocyte-mediated effect cannot be excluded, based on the recent finding that SR101 labels oligodendrocytes *in vivo*^6^ and the intimate proximity between neuronal axon initial segments and myelin sheathes^29^. Overall, the current knowledge of SR101-evoked changes of neuronal excitability and its uptake time course leads us to suggest, for now, that the most likely mechanism of action is via a direct effect on neurones, but future experiments should aim to answer this question in more detail.

The main purpose of applying SR101 to the cortical surface before initiating 2-photon microscopy experiments, is to label astrocytes in order to unambiguously distinguishing between neuronal and astrocytic signaling *in vivo*^1^,^4^,^10^,^30^. Thus, any interference with normal brain physiology mediated by SR101 is in that context strongly undesirable. Although, we have not explored the after-effects of SR101-induced seizure-like LFP activity and its influence on subsequent experiments, other studies indicate that some long-term effects could be expected. In our earlier work we showed that ten minutes of SR101 bath application evoke long-term potentiation of synaptic efficacy lasting 80 minutes following exposure in hippocampal slices^9^. Similar results have been reported by Garaschuk showing increased population spike amplitude 50 minutes following SR101 exposure^10^. Overall this suggests, that the exposure of the neuronal tissue to SR101 can cause long-term changes in synaptic efficacy of excitatory transmission. In addition, previous observations show that epileptic seizure activity can induce long-term synaptic plasticity^31^,^32^, as well as increase the expression of the synaptic plasticity-associated immediate-early gene c-Fos^33^,^34^. Finally, recent work shows that spontaneous interictal epileptiform discharges can impair memory consolidation^18^. Hence, it is a possiblity that the observed 30–40 minutes of abnormal cortical LFP activity (Figs 1 and 2) cause similar changes in synaptic plasticity, and impacts subsequent physiological recordings, such as experiments investigating synaptic plasticity *in vivo*. In agreement with this consideration is previous work discussing spontaneous Ca^2+^ transients in visual cortex before and after injection of 100 μM SR101 (described in)^10^. Here the authors described that SR101 injection initially caused an increase in the frequency of Ca^2+^ transients, supporting our LFP observations (Figs 1 and 2). However, 40 minutes after the SR101 injection spontaneous Ca^2+^ transients were significantly reduced compared to baseline, suggestive of long-term depression. Thus, overall, SR101 is able to change cortical synaptic efficacy, on a time scale that impact experimental data obtained *in vivo*.

SR101 was originally discovered to selectively label cortical astrocytes in 2004^8^. Thus, the question arises why the potent effects of SR101 concentrations ≥100 μM on cortical LFP activity have not been reported until now. As previously described, the main purpose of SR101 application is to label astrocytes to distinguish between neuronal- and astrocytic Ca^2+^ transients. For measuring Ca^2+^ signals organic Ca^2+^ indicator dyes, such as Oregon Green 488 BAPTA-1 AM, has been widely used^1^,^4^,^8^,^30^. This Ca^2+^ indicator dye require ~1 hour following loading to reach a stable maximal fluorescence level in stained cells^30^. In our experiments, we observed that SR101-triggered seizure-like LFP activity ceased after 30–40 minutes, depending on the SR101 concentration used (Fig. 1). Thus, if SR101 is applied together with an organic Ca^2+^ indicator dye, and the subsequent experiment is not initiated until 1 hour following loading, the abnormal neuronal activity is likely not seen. Furthermore, in our experiments, we applied SR101 onto S1-M1 cortical areas, allowing us to observe behavioural outputs, *i.e.* contra-lateral hindlimb muscle contractions, during SR101-induced seizure-like LFP activity (Fig. 1, Supplementary Fig. S4 and Supplementary Videos S1–3). However, if SR101 is applied to non-motor cortical areas, *e.g.* visual- or auditory cortex, no such obvious behavioural outputs might develop, again masking the abnormal neuronal activity from the experimenter.

In summary, we here show that commonly used loading concentrations of the astrocyte-specific indicator SR101, *i.e.* 100 μM and 250 μM, can induce seizure-like activity in S1-M1 five to ten minutes following topical loading, when incubated for ten minutes. This SR101-induced synchronized cortical activity can evoke motor outputs and muscle contractions even in anaesthetised mice. Finally we show, that 25 μM and 50 μM SR101 does not appear to cause abnormal LFP activity following loading, but still labels cortical astrocytes. Overall, our observations may call into question the widespread usage of 100–300 μM SR101 for topical loading of cortical astrocytes, given its potent bioactive effects on cortical tissue when incubated for ten minutes. We advocate that SR101 concentrations no higher than 50 μM should be used for *in vivo* experiments, to avoid the risk of evoking abnormal cortical neuronal activity.

## Methods

### Animal preparation

All experiments carried out in Rochester were in compliance with the National Institute of Health Guide for the Care and Use of Laboratory Animals and in accordance with guidelines approved by the institute ethics committee for the care and use of animals. All experiments performed at the University of Copenhagen were approved by the Danish National Animal Experiment Committee (Permission No. 2012-15-2934-00719 and 2015-15-0201-00535) and was in accordance with European Union Regulations.

Mouse preparation was modified from previously published protocol^35^. Wild-type mice of either sex, 6–12 weeks old (N = 30), were anaesthetised with an intraperitoneal injection of ketamine (120 mg kg–1) and xylazine (10 mg kg–1) (KX) dissolved in saline. Depth of anaesthesia was monitored by pinch withdrawal reflex throughout the experiment. We supplied half the initial dose of KX to the anaesthesia every 60 minutes. If the mouse responded to pinch stimulation additional KX anaesthesia was given immediately. Core body temperature was monitored by a rectal probe and maintained at 37 ± 0.5 °C by a heating blanket (BS4, Harvard Apparatus). Eyes were protected with eye ointment (Stye, NY USA). A custom-made metal plate was glued to the exposed and cleaned skull with cyanoacrylate. A craniotomy (2–3 mm in diameter), with the centre 0.5 mm posterior to bregma and 3.5 mm lateral from the midline, was prepared over the right or left primary sensori-motor area (S1-M1) of the cortex and the dura carefully removed while necessary care was taken to avoid damage the cortical tissue (Fig. 1a). The cortical surface was kept moist by topical Ringer solution application (in mM: 126 NaCl, 3.5 KCl, 1.24 NaH2PO4, 26 NaHCO3, 10 glucose, 2 CaCl2 and 1 MgSO4 at pH 7.4). In a subset of experiments, the contra-lateral sciatic nerve was exposed and dissected free from the surrounding tissues (similar to)^36^, cut distally and then mounted on a custom-made hook-electrode (Fig. 1a). We used this arrangement for recording of compound action potentials in the motor axons projecting to skeletal muscles of the distal hindlimb, providing us a measure of the timing of cortical output compared with the peripheral motor activity. The skin surrounding the exposed muscles and nerves of the hindlimb was sewed to a metal frame forming a bath that was filled with warm paraffin oil to reduce current shunting to the bathing media. For the experiments in awake mice (Fig. 2a) we adapted a published protocol^23^. Briefly, mice were anaesthetised using KX, head restrained with a custom-made mini-frame glued to the skull with cyanoacrylate and habituated to the restraint over two days, with a total training duration of 3–4 hours. On the day of the experiment a craniotomy was prepared as described above in isoflurane anesthesia, and the mice were allowed to recover from the anaesthesia for 1 hour prior to conducting the experiments in order to avoid any after-effects on cortical activity. The mice were during recordings placed in a behavioural tube to minimise movement and to comfort the mice (Fig. 2a)^23^, and the room was kept dark and quiet. Body temperature was maintained as described above.

### Electrophysiology

We recorded primary sensori-motor cortical layer II (100–150 μm below the pial surface) local field potentials (LFP) continuously using a NaCl-filled glass micro-electrode (Resistance 2–5 MΩ) connected to a DP-311 differential amplifier (Warner Instruments, Hamden, CT, USA). Filters were set at 0.1 Hz high-pass and 100 Hz low-pass and sampled at 10 kHz using a power1401 digitiser (Cambridge Electronic Design, UK) and Spike2 version 6 (CED, UK) or pCLAMP 9.2 software (Axon Instruments, Sunnyvale, CA, USA). The electroneurogram (ENG) was recorded with a custom-made hook-electrode (Fig. 1a) connected to a DP-311 differential amplifier (Warner instruments, Hamden, USA) and high-pass and low-pass filtered at 1 Hz and 1 kHz, respectively. The ENG signal was digitised and sampled at 2 kHz using a power1401 digitizer (Cambridge Electronic Design, UK) and Spike2 version 6 software (CED, UK).

### 2-photon *in vivo* microscopy

For visualising sulforhodamine 101 (SR101) uptake into cortical astrocytes *in vivo* (Figs 1b and 3b) we used 2-photon microscopy as previously described^23^. We used a custom-built (modified Olympus Fluoview 300) microscope (Olympus software Fluoview 300) with a 20× water-immersion objective (NA 0.95, XLUMPLFL20XW, Olympus), linked to a Ti:sapphire laser (Mai Tai, Spectra-Physics). Excitation wavelength was set at 800 nm. The emitted light was separated with an emission filter (ET595/60 Chroma). The laser power going into the cortical tissue was in the range of 20–30 mW^37^.

### Dye application

SR101 (Molecular Probes, S359) was dissolved in Ringer solution to concentrations of 25 μM, 50 μM, 100 μM or 250 μM, all within the range for what is used in the litterature^4^,^15^,^19^,^20^ (see Supplementary Table S1). In some experiments we tested Texas Red (Dextran, Molecular Probes, D1863), a tissue fixable analog of SR101, at concentrations of 100 μM or 500 μM. The dissolved dye was topically applied to the exposed dura-free cortical surface. Right before application of the dye, the Ringer solution bathing the craniotomy was carefully absorbed. The dye solution was then applied using a pipette onto the exposed cortex to cover the cranial window. The SR101 was kept on the cortical surface for 1, 10, 20 or 30 min, depending on the concentration used, all incubation durations were within the range for what is used in the literature (see Supplementary Table S1), and carefully washed away using Ringer solution (see Supplementary Fig. S3). Only one SR101 concentration was used in each experiment. In four experiments we pressure ejected the SR101 (1 min pulse, 120–200 mbar; 100 μM SR101) onto the cortical surface under agarose (1.5%, Sigma) and a glass coverslip. This approach enabled us to image the time course of astrocytic SR101 uptake using 2-photon microscopy (Fig. 1b). To eliminate the possibility that our experimental observations resulted from a contaminated batch of SR101 we used several different SR101 batches over the course of many months, all were bought from Molecular Probes.

### LFP analysis

#### Power measurements

LFP processing and analysis was performed using MATLAB (2015a, The MathWorks, Massachusetts, United States). First, any 50 Hz or 60 Hz line noise were removed from the recording using the *rmlinesc* function, available from the Chronux 2.0, an open-source, MATLAB-based, data analysis toolbox available at http://chronux.org/38. The de-noised LFP was then down sampled to 1kHz using the *resample* function in MATLAB. LFP traces chosen for power spectral analysis was then normalized to the z-score^18^ by subtracting the mean and dividing by the standard deviation of the LFP during the entire LFP recording. For exploring the frequency-domain dynamics in LFPs we used the *mtspectrumc* function, a multitaper method implemented in the Chronux 2.0 toolbox^38^. For this analysis we used a padding factor of -1, time-bandwidth product of 3 and 5 tapers. For the frequency-domain graphs (Fig. 2c and Supplementary Fig. S1) a moving average and moving standard deviation with a window size of 50 was applied afterwards. For Supplementary Fig. S7 no moving average or moving standard deviation was applied, in order to show the raw frequency-domain dyamics. For Supplementary Fig. S1f the average power (in decibel) was determined for delta (1–4 Hz) and beta (12–30 Hz) LFP bands within period 1 (*i.e.* baseline) and period 2 (*i.e.* SR101-induced LFP activity) for both anaesthetised and awake recordings. The relative change in power density (*i.e.* period 2 vs period 1, reported in % of baseline) was then determined for both anaesthetised and awake recording. Time-frequency spectrograms were constructed using the multitaper method implemented in the *mtspecgramc* function in the Chronux 2.0 toolbox^38^. For this analysis we used a padding factor of −1, a moving window of 2.5 s in duration with a slide of 50 ms along the time axis and time-bandwidth product of 3 and 5 tapers. All power measurements are reported in units of decibel (dB) unless otherwise stated.

#### Event detection

For determining the individual event amplitude and event intervals for the synchronized cortical seizure-like activity (Figs 1d and 2e) we used custom-made procedures in MATLAB. First a threshold was determined as 4 standard deviations (S.D.) more negative than the mean LFP during baseline (first 5 min of recording) and synchronized cortical seizure-like events were located using this threshold. Next the amplitude and event interval was determined for all of the events in the recording. Amplitudes and event intervals across experiments were pooled and histograms produced using the *histogram* function with 40 bins in MATLAB. We determined the synchronized cortical seizure-like activity onset and cessation relative to topical application of either 100 μM or 250 μM SR101 (Fig. 1e and Supplementary Fig. S2) using custom-made procedures in MATLAB. For this we determined time-points for the first and last seizure-like event in a recording using a threshold determined as 4 standard deviations more negative than the mean LFP during baseline (first 5 min of recording) and calculated the delta times from the time-point of SR101 application.

#### Correlation between LFP and ENG events

For determining the correlation between seizure-like events and ENG compound action potential events (Fig. 1f and Supplementary Fig. S4) we first determined the time-points for all the seizure-like events in a recording and converted the LFP recording into binaries, *i.e.* 0 if no seizure-like event and 1 if seizure-like event was present, using a threshold determined as 4 S.D. more negative than the mean LFP during baseline (first 5 min of recording). Next we screened the ENG recording for the presence of compound action potentials, using a threshold as 4 S.D. from the mean ENG during baseline (first 5 min of recording), in the time windows corresponding to where the binary LFP was 1. If ENG compound action potentials were present in the time window, that window was assigned 1 and if no compound action potentials were present assigned 0. Afterwards, we plotted the cumulative number of seizure-like LFP events versus the cumulative number of ENG compound action potential events at those time points and calculated the linear correlation using a Pearsons’s correlation test (Fig. 1f).

### Statistics

Our preliminary experiments suggested the existence of very large LFP changes from topical SR101 application. We therefore used the smallest number of mice that is neccesary to compare statistically based on power analysis considering that all mice exposed to 100 μM and 250 μM SR101 for 10 min developed seizure-like activity, which was not observed in any control experiments (see Supplementary Fig. S2). Statistical comparison between relative onsets- and cessation of seizure-like activity between 100 μM and 250 μM SR101 was assessed using a two-sided Welch’s *t*-test (Fig. 1d). This test was chosen due to the violation of the Student’s *t*-test assumptions, *i.e.* equal variances and sample sizes^39^. Statistical comparision for power spectral density in 1–4 Hz and 12–30 Hz LFP bands during baseline compared to during SR101-induced seizure-like activity was assessed using a paired Student’s *t*-test. We considered significant tests with *P* < 0.05. Exact *P*-values are reported unless smaller than 0.0001. For calculating the linear correlation between the cumulative number of seizure-like LFP events versus the cumulative number of ENG compound action potential events we used the Pearsons’s correlation test and reported the R^2^-value (Fig. 1e). Data is plotted as mean ± S.D. where appropriate. Figures were created with Illustrator CC 19.2.1 (Adobe, USA).

## Additional Information

**How to cite this article**: Rasmussen, R. *et al.* Sulforhodamine 101, a widely used astrocyte marker, can induce cortical seizure-like activity at concentrations commonly used. *Sci. Rep.* **6**, 30433; doi: 10.1038/srep30433 (2016).

## Supplementary Material

Supplementary Video 1

Supplementary Video 2

Supplementary Video 3

Supplementary Information
