# Supplementary material for: Sulforhodamine 101, a widely used astrocyte marker, can induce cortical seizure-like activity at concentrations commonly used
Source: Sci Rep. 2016 Jul 26;6:30433. doi: 10.1038/srep30433 (PMC4960645; doi:10.1038/srep30433)
Supplement: Supplementary Information [file srep30433-s4.pdf]

SUPPLEMENTARY MATERIAL

Sulforhodamine 101, a widely used astrocyte marker, can induce cortical seizure-like neuronal activity at concentrations commonly used

Rune Rasmussen[1,4], Maiken Nedergaard[1,4] and Nicolas Caesar Petersen[2,3,*]

[1]Center for Translational Neuromedicine, University of Rochester Medical Center, Rochester, New York 14642. [2]Department of Nutrition, Exercise and Sports, University of Copenhagen, 2200 Copenhagen N, Denmark. [3]Department of Neuroscience and Pharmacology, University of Copenhagen, 2200 Copenhagen N, Denmark. [4]Center for Basic and Translational Neuroscience, University of Copenhagen Faculty of Medicine, 2200 Copenhagen N, Denmark.

Supplementary File	Title
**Supplementary Figure S1**	Power spectral analysis of SR101-evoked seizure-like activity
**Supplementary Figure S2**	Control LFP recording in mouse primary sensori-motor cortex
**Supplementary Figure S3**	Non-filtered LFP recording showing cortical SR101-evoked seizure-like activity
**Supplementary Figure S4**	Simultaneous LFP and ENG recording during SR101-evoked cortical seizure-like activity
**Supplementary Figure S5**	25 µM or 50 µM SR101 does not evoke seizure-like activity in mouse sensori-motor cortex
**Supplementary Figure S6**	100 µM or 500 µM Texas Red does not evoke seizure-like activity in sensori-motor cortex
**Supplementary Figure S7**	LFP frequency power distribution change between anaesthetized and awake mouse cortex
**Supplementary Video S1**	100 µM SR101-evoked muscle contractions in contra-lateral hindlimb
**Supplementary Video S2**	250 µM SR101-evoked muscle contractions in contra-lateral hindlimb
**Supplementary Video S3**	250 µM SR101-evoked muscle contractions with hindlimb pinch
**Supplementary Table S1**	Overview of SR101 usage in vivo

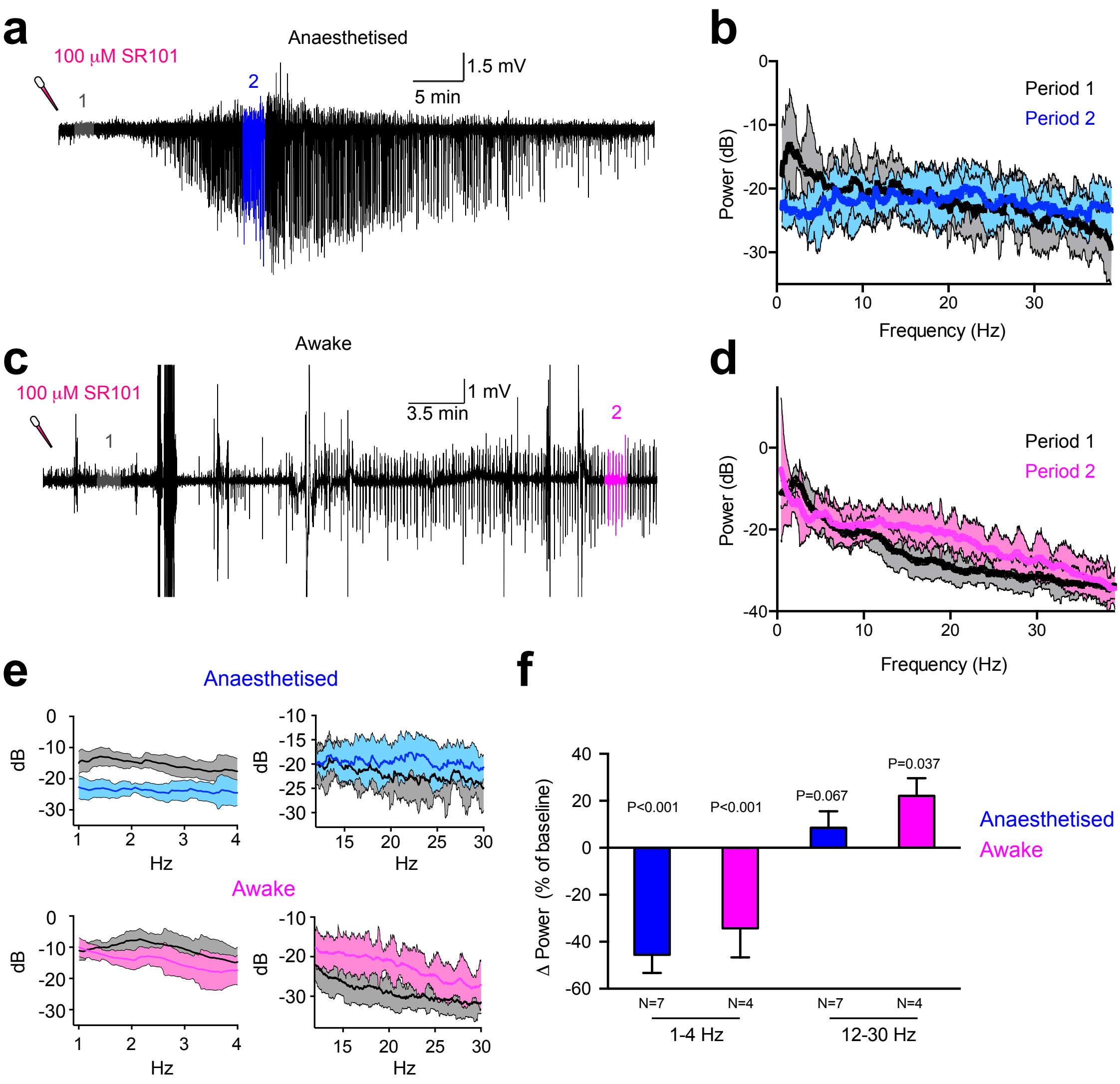

Supplementary Figure S1. Power spectral analysis of SR101-evoked seizure-like activity. **(a)** LFP recording in anaesthetized mouse showing development of seizure-like activity in primary sensori-motor cortex following 100 µM SR101 loading (10 min incubation). The grey and blue coloured LFP epochs, period 1 and 2 respectively, was used for for power spectral analysis. **(b)** Frequency-domain power spectra showing change in power distribution between normal LFP activity (Period 1) and SR101-evoked seizure-like activity (Period 2) in anaesthetized mouse. **(c)** LFP recording in awake mouse showing development of seizure-like activity in primary sensori-motor cortex following 100 µM SR101 loading (10 min incubation). The grey and magenta coloured LFP epochs, period 1 and 2 respectively, was used for for power spectral analysis. Note that movement artefacts has not been removed from the LFP. **(d)** Frequency-domain power spectra showing change in power distribution between normal LFP activity (Period 1) and SR101-evoked seizure-like activity (Period 2) in awake mouse. (**e**) Graphs showing close-up differences in frequency-domain power spectra (left, 1-4 Hz and right, 12-30 Hz) during SR101-induced seizure-like activity in anaesthetised and awake mice. (**f**) Relative changes in power density (% of baseline) for 1-4 Hz and 12-30 Hz LFP bands during SR101-induced seizure-like activity in anaesthetised and awake mice. Statistical difference was assessed using a paired Student's *t*-test.

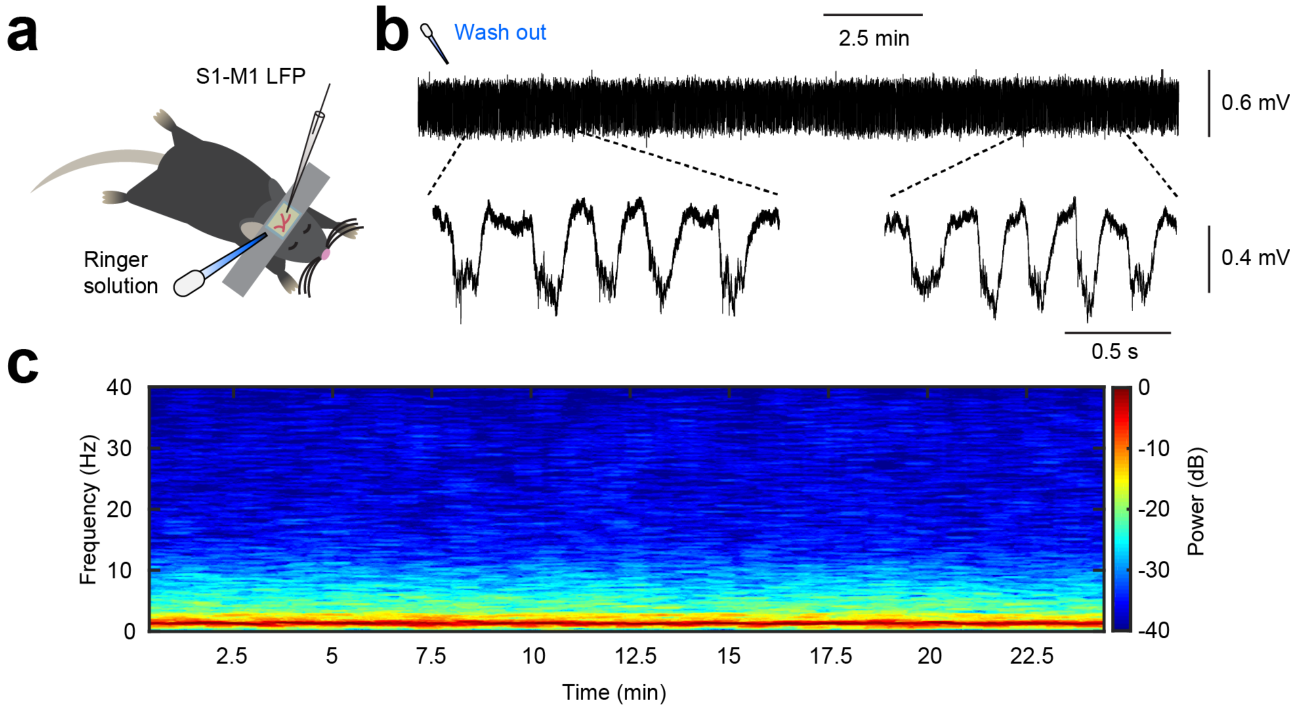

Supplementary Figure S2. Control LFP recording in primary sensori-motor cortex. (**a**) Experimental setup showing LFP recording in primary sensori-motor cortex (S1-M1) and control wash out using Ringer solution. (**b**) Full LFP recording during and following control wash out using Ringer solution in anaesthetized mouse. (**c**) Spectrogram showing frequency power distribution in the LFP recording shown in (**b**).

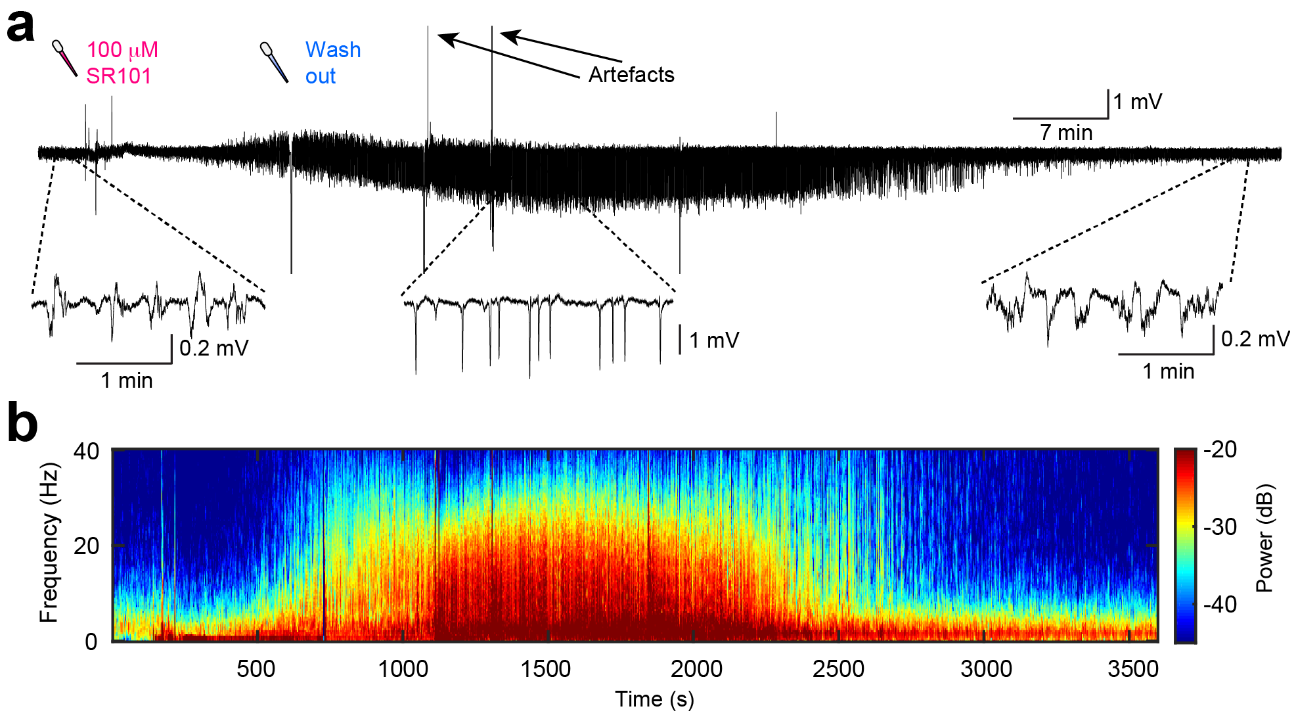

Supplementary Figure S3. Non-filtered LFP recording showing SR101-evoked seizure-like activity. (**a**) LFP recording in anaesthetized mouse showing development of seizure-like activity in primary sensori-motor cortex following 100 µM SR101 loading (10 min incubation). The LFP trace was not filtered and does thus contain the artefacts from topical SR101 loading and wash out 10 min later using Ringer solution. Please note that the artefacts marked with arrows does not represent SR101 wash out, but two electrical interference artefacts. (**b**) Spectrogram showing frequency power distribution in the non-filtered LFP recording shown in (**a**).

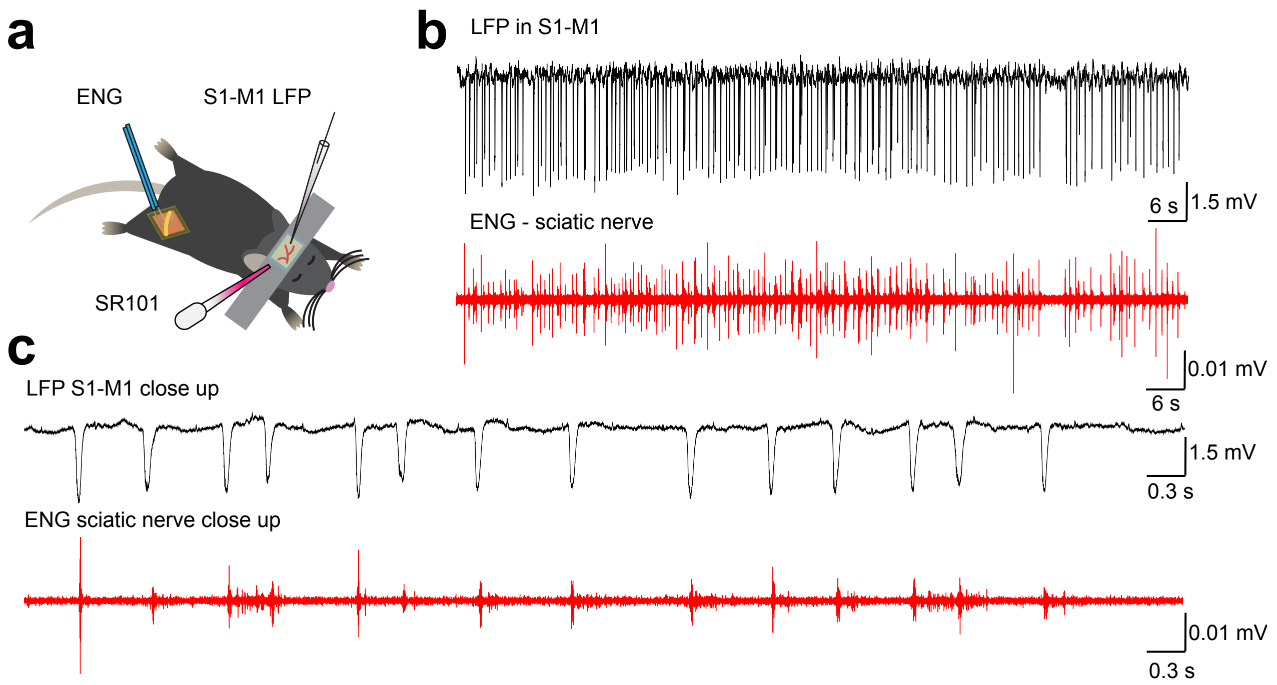

Supplementary Figure S4. Simultaneous LFP and ENG recording during SR101-evoked cortical seizure-like activity. (**a**) Experimental setup showing LFP recording in primary sensori-motor cortex (S1-M1) and simultaneous electroneurogram (ENG) recording in contra-lateral hindlimb sciatic nerve in anaesthetized mouse. (**b**) Simultaneous LFP recording in S1-M1 and ENG recording from sciatic nerve 20 min following 250 µM SR101 loading (10 min incubation). (**c**) Close up of the LFP and ENG recording shown in (**c**). Note the time-locked relationship between the events in the two voltage signals.

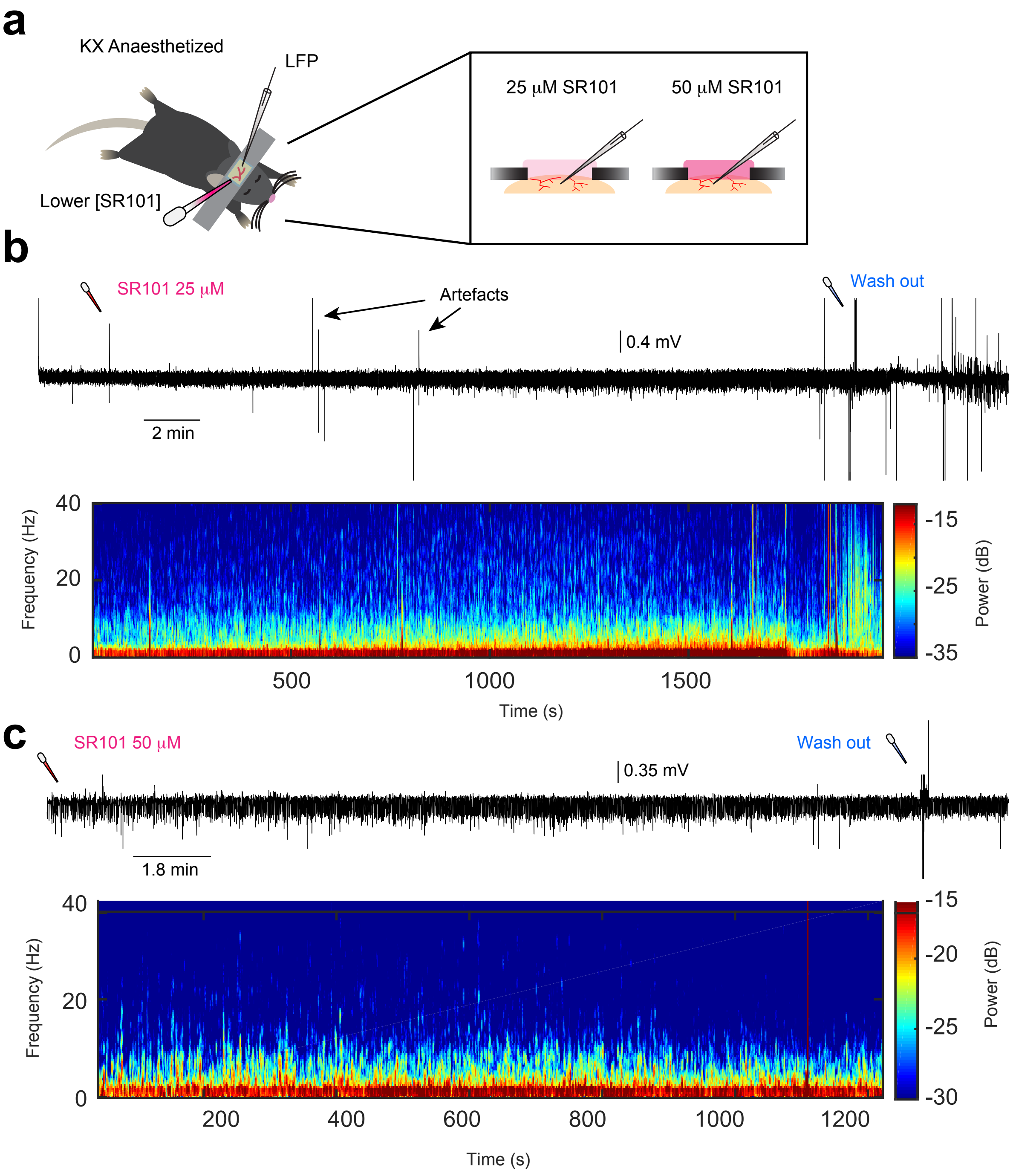

Supplementary Figure S5. 25 µM or 50 µM SR101 does not evoke seizure-like activity in primary sensori-motor cortex. (**a**) Experimental setup showing LFP recording in primary sensori-motor cortex in anaesthetized mouse. Note that only one concentration of SR101 (*i.e.* either 25 µM or 50 µM) was used in each experiments. (**b**) Upper, LFP recording showing loading of 25 µM SR101 and wash out 30 minutes later. Lower, spectrogram showing frequency power distribution in the LFP recording shown above. (**c**) Upper, LFP recording showing loading of 50 µM SR101 and wash out 20 min later. Lower, spectrogram showing frequency power distribution in the LFP recording shown above.

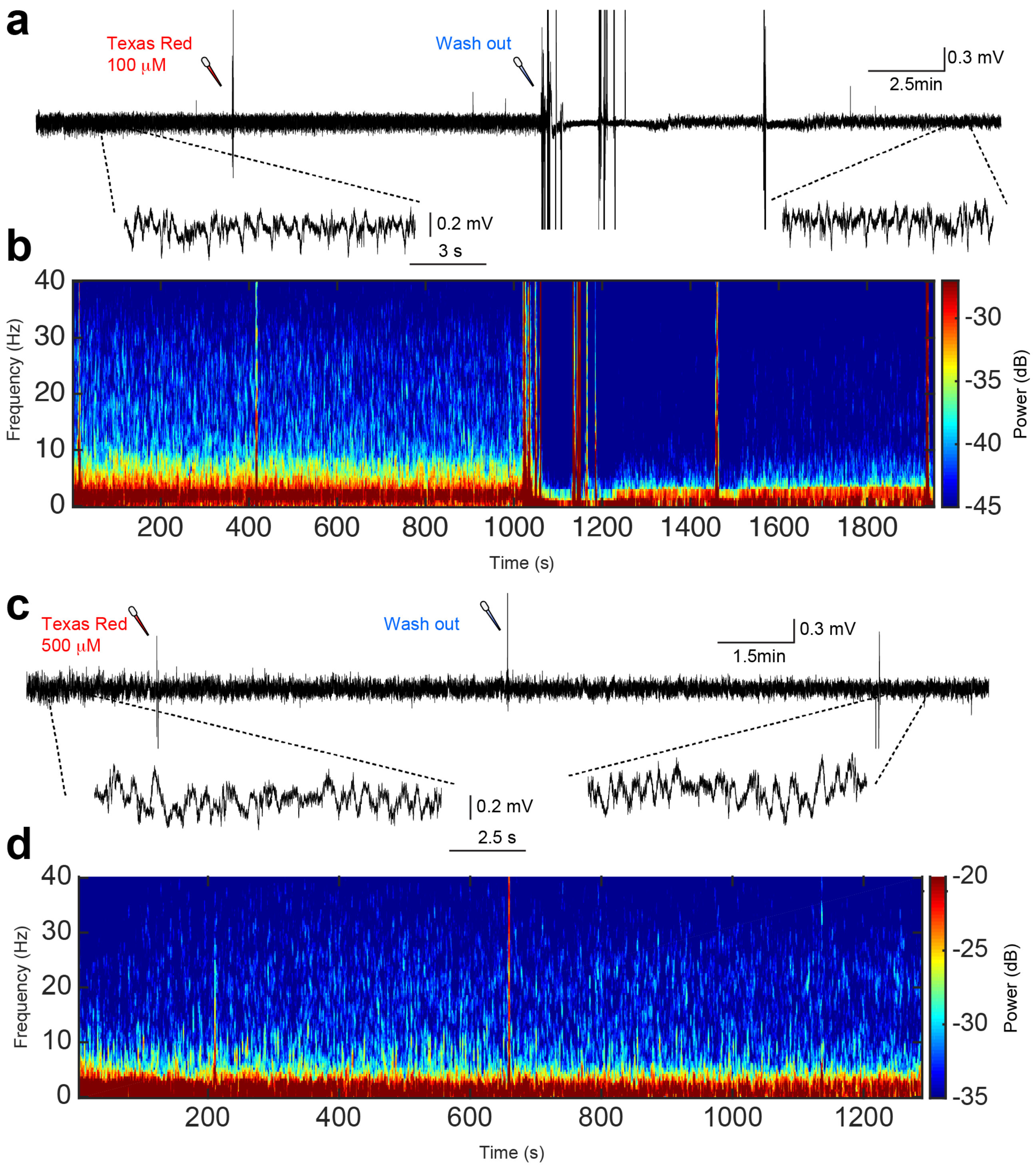

Supplementary Figure S6. Texas Red does not evoke seizure-like activity in primary sensori-motor cortex. (**a**) LFP recording showing loading of 100 μM Texas Red and wash out 15 min later. (**b**) Spectrogram showing frequency power distribution in the LFP recording shown in (**a**). (**c**) LFP recording showing loading of 500 μM Texas Red and wash out 10 min later. (**d**) Spectrogram showing frequency power distribution in the LFP recording shown in (**c**).

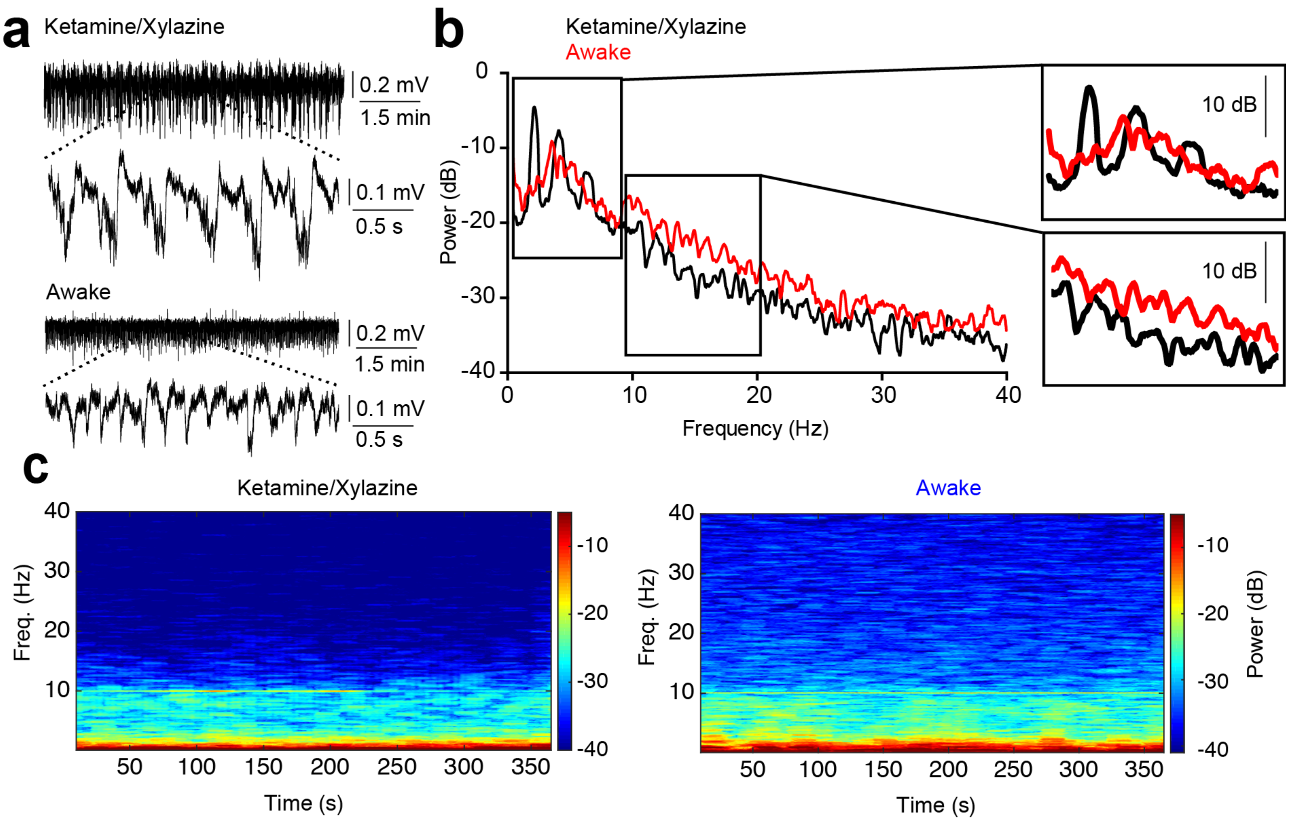

Supplementary Figure S7. LFP Frequency power distribution change between anaesthetized and awake mouse. (**a**) LFP recording epochs during ketamine/xylazine (KX) anaesthesia or wakefulness in the same mouse. (**b**) Frequency-domain spectra showing change in power distribution between KX anaesthesia (black) and awake (red) LFP shown in (**a**). Note the drop in power around 2-3 Hz and increase in power around 10-20 Hz in the awake LFP recording compared to the KX LFP. No moving average or moving standard deviation was applied to the traces, in order to show raw frequency-domain power spectra traces. (**c**) Spectrograms showing frequency power distribution in the KX (Left) and awake (Right) LFP recording, respectively, shown in (**a**).

Supplementary Video S1. 100 μM SR101-evoked muscle contractions in contra-lateral hindlimb. Video showing muscle contractions in contra-lateral hindlimb 20 min following 100 μM SR101 topical loading above the left primary sensori-motor cortex in ketamine/ xylazine anaesthetized mouse. Note that the muscle contractions are selectively occurring in the contra-lateral side.

Supplementary Video S2. 250 μM SR101-evoked muscle contractions in contra-lateral hindlimb. Video showing muscle contractions in contra-lateral hindlimb 17 min following 250 μM SR101 topical loading above the left primary sensori-motor cortex in ketamine/ xylazine anaesthetized mouse. Note that the muscle contractions are selectively occurring in the contra-lateral side.

Supplementary Video S3. 250 μM SR101-evoked muscle contractions in contra-lateral hindlimb. Video showing muscle contractions in contra-lateral hindlimb 20 min following 250 μM SR101 topical loading above the right primary sensori-motor cortex in ketamine/ xylazine anaesthetized mouse. Note that the muscle contractions are selectively occurring in the contra-lateral side and that the mouse does not respond to the pinch stimulation on the ipsi-lateral side, proving the depth of the anaesthesia and lack of reflex response in the hindlimb.

Supplementary Table S1 - Overview of SR101 usage *in vivo*

Application Route	Final Concentration	Incubation Duration	Dura matter removed	Reference
Topical	1 µM	10 min	Yes	1
Topical	40 µM	30 min	Yes	2
Topical	50 µM	20 min	Yes	3
Topical	50 µM	5-10 min	Yes	4
Topical	50 µM	5-10 min	Not described	5
Topical	25-100 µM	1-5 min	Yes (in rats)	6
Topical	100 µM	10 min	Yes	7
Topical	100 µM	1-5 min	Yes	8
Topical	100 µM	30 min	Yes	9
Topical	100 µM	1-5 min	Yes	10
Topical	100 µM	45 min	Yes	11
Topical	100 µM	3 min	Yes	12
Topical	100 µM	20 min	Yes	13
Topical	125 µM	30-60 min	Not described	14
Topical	200 µM	30 min	No	15
Topical	200-333 µM	1-5 min	Yes	16
Topical	200-300 µM	3-5 min	No	17
Topical	412 µM	30 min	Yes	18
Topical	1 mM	Not described	Yes	19
Topical	1 mM	Not described	Yes	20

*Continued on next page

Application Route	Final Concentration	Incubation Duration	Dura matter removed	Reference
Cortical injection	25-100 μM	-	-	6
Cortical injection	100 μM	-	-	21
Cortical injection	100 μM	-	-	22
Cortical injection	100 μM	-	-	23
Cortical injection	100 μM	-	-	24
Cortical injection	100 μM	-	-	25
Cortical injection	100 μM	-	-	26
Cortical injection	200 μM	-	-	15
Cortical injection	200-333 μM	-	-	16
Cortical injection	200-500 μM	-	-	17
Cortical injection	412 μM	-	-	27
Cortical injection	1 mM	-	-	28
Cortical injection	40 mM	-	-	29

Supplementary Table 1. Table showing how SR101 is used for *in vivo* experiments. Information include application route (*i.e.* topical or cortical injection), SR101 concentration applied, and when stated, the incubation duration before wash and whether the dura matter was removed (only applies for topical application). Publications were selected from a literature search on PubMed using the key-word "Sulforhodamine 101", and publications using SR101 for *in vivo* cortical labelling were included in the table. We did not include experiments using intra-peritoneal[30] or intra-venous[31] injection of SR101 as this method is less common than the topical and cortical injection routes.

References

1. Chuquet, J., Quilichini, P., Nimchinsky, E. A. & Buzsáki, G. Predominant enhancement of glucose uptake in astrocytes versus neurons during activation of the somatosensory cortex. *J. Neurosci.* **30,** 15298–303 (2010).
2. Cayce, J. M. *et al.* Calcium imaging of infrared-stimulated activity in rodent brain. *Cell Calcium* **55,** 183–90 (2014).
3. Schain, A. J., Hill, R. A. & Grutzendler, J. Label-free in vivo imaging of myelinated axons in health and disease with spectral confocal reflectance microscopy. *Nat. Med.* **20,** 443–9 (2014).
4. Langer, D. & Helmchen, F. Post hoc immunostaining of GABAergic neuronal subtypes following in vivo two-photon calcium imaging in mouse neocortex. *Pflügers Arch. Eur. J. Physiol.* **463,** 339–54 (2012).
5. Hill, R. A. & Grutzendler, J. In vivo imaging of oligodendrocytes with sulforhodamine 101. *Nat. Methods* **11,** 1081–2 (2014).
6. Nimmerjahn, A., Kirchhoff, F., Kerr, J. N. D. & Helmchen, F. Sulforhodamine 101 as a specific marker of astroglia in the neocortex in vivo. *Nat. Methods* **1,** 31–37 (2004).
7. Tian, G.-F. *et al.* An astrocytic basis of epilepsy. *Nat. Med.* **11,** 973–81 (2005).
8. Ding, S. *et al.* Enhanced astrocytic Ca2+ signals contribute to neuronal excitotoxicity after status epilepticus. *J. Neurosci.* **27,** 10674–84 (2007).
9. Haiss, F. *et al.* Improved in vivo two-photon imaging after blood replacement by perfluorocarbon. *J. Physiol.* **587,** 3153–8 (2009).
10. Ding, S., Wang, T., Cui, W. & Haydon, P. G. Photothrombosis ischemia stimulates a sustained astrocytic Ca2+ signaling in vivo. *Glia* **57,** 767–76 (2009).
11. Wang, X. *et al.* Astrocytic Ca2+ signaling evoked by sensory stimulation in vivo. *Nat. Neurosci.* **9,** 816–23 (2006).
12. Karatas, H. *et al.* Spreading depression triggers headache by activating neuronal Panx1 channels. *Science* **339,** 1092–5 (2013).
13. Kim, S. K. *et al.* Cortical astrocytes rewire somatosensory cortical circuits for peripheral neuropathic pain. *J. Clin. Invest.* (2016).
14. Navarrete, M. *et al.* Astrocytes mediate in vivo cholinergic-induced synaptic plasticity. *PLoS Biol.* **10,** e1001259 (2012).
15. McCaslin, A. F. H., Chen, B. R., Radosevich, A. J., Cauli, B. & Hillman, E. M. C. In vivo 3D morphology of astrocyte-vasculature interactions in the somatosensory cortex: implications for neurovascular coupling. *J. Cereb. Blood Flow Metab.* **31,** 795–806 (2011).

16. Nimmerjahn, A., Mukamel, E. A. & Schnitzer, M. J. Motor behavior activates Bergmann glial networks. *Neuron* **62,** 400–12 (2009).
17. Nimmerjahn, A. & Helmchen, F. In vivo labeling of cortical astrocytes with sulforhodamine 101 (SR101). *Cold Spring Harb. Protoc.* **7,** 326–334 (2012).
18. Galea, E. *et al.* Topological analyses in APP/PS1 mice reveal that astrocytes do not migrate to amyloid-β plaques. *Proc. Natl. Acad. Sci. U. S. A.* **112,** 15556–61 (2015).
19. Jessen, S. B. *et al.* GABAA Receptor-Mediated Bidirectional Control of Synaptic Activity, Intracellular Ca2+, Cerebral Blood Flow, and Oxygen Consumption in Mouse Somatosensory Cortex In Vivo. *Cereb. Cortex* **25,** 2594–609 (2015).
20. Lind, B. L., Brazhe, A. R., Jessen, S. B., Tan, F. C. C. & Lauritzen, M. J. Rapid stimulus-evoked astrocyte Ca2+ elevations and hemodynamic responses in mouse somatosensory cortex in vivo. *Proc. Natl. Acad. Sci. U. S. A.* **110,** E4678–87 (2013).
21. Delekate, A. *et al.* Metabotropic P2Y1 receptor signalling mediates astrocytic hyperactivity in vivo in an Alzheimer's disease mouse model. *Nat. Commun.* **5,** 5422 (2014).
22. Sohya, K., Kameyama, K., Yanagawa, Y., Obata, K. & Tsumoto, T. GABAergic neurons are less selective to stimulus orientation than excitatory neurons in layer II/III of visual cortex, as revealed by in vivo functional Ca2+ imaging in transgenic mice. *J. Neurosci.* **27,** 2145–9 (2007).
23. Ishikawa, D., Matsumoto, N., Sakaguchi, T., Matsuki, N. & Ikegaya, Y. Operant conditioning of synaptic and spiking activity patterns in single hippocampal neurons. *J. Neurosci.* **34,** 5044–53 (2014).
24. Rikhye, R. V & Sur, M. Spatial Correlations in Natural Scenes Modulate Response Reliability in Mouse Visual Cortex. *J. Neurosci.* **35,** 14661–80 (2015).
25. Zhao, J., Wang, D. & Wang, J.-H. Barrel cortical neurons and astrocytes coordinately respond to an increased whisker stimulus frequency. *Mol. Brain* **5,** 12 (2012).
26. Chen, N. *et al.* Nucleus basalis-enabled stimulus-specific plasticity in the visual cortex is mediated by astrocytes. *Proc. Natl. Acad. Sci. U. S. A.* **109,** E2832–41 (2012).
27. Garaschuk, O., Milos, R.-I. & Konnerth, A. Targeted bulk-loading of fluorescent indicators for two-photon brain imaging in vivo. *Nat. Protoc.* **1,** 380–6 (2006).
28. Takata, N. & Hirase, H. Cortical layer 1 and layer 2/3 astrocytes exhibit distinct calcium dynamics in vivo. *PLoS One* **3,** e2525 (2008).

29. Hagihara, K. M. & Ohki, K. Long-term down-regulation of GABA decreases orientation selectivity without affecting direction selectivity in mouse primary visual cortex. *Front. Neural Circuits* **7,** 28 (2013).
30. Appaix, F. *et al.* Specific in vivo staining of astrocytes in the whole brain after intravenous injection of sulforhodamine dyes. *PLoS One* **7,** (2012).
31. Hill, R. A. & Grutzendler, J. In vivo imaging of oligodendrocytes with sulforhodamine 101. *Nat. Methods* **11,** 1081–2 (2014).
